# Climate variability and trends at a national scale

**DOI:** 10.1038/s41598-017-03297-5

**Published:** 2017-06-12

**Authors:** Zhenci Xu, Ying Tang, Thomas Connor, Dapeng Li, Yunkai Li, Jianguo Liu

**Affiliations:** 1 Center for Systems Integration and Sustainability, Department of Fisheries and Wildlife, Michigan State University, East Lansing, 48823 USA; 2 College of Water Resources and Civil Engineering, China Agricultural University, Beijing, 100083 China; 3 Department of Geography, Environment, and Spatial Sciences, Michigan State University, East Lansing, 48823 USA

## Abstract

Climate variability and trends have significant environmental and socioeconomic impacts. Global challenges such as food security, biodiversity loss, water scarcity and human health are affected by reference evapotranspiration, temperature, solar radiation, and precipitation together, but nonlinear dynamics of these four climatic factors have not been assessed simultaneously at the national scale. This leads to unclear climatic dynamics and limited applications. To address this knowledge gap, we analyzed the daily variability and trends of four climatic factors (reference evapotranspiration, temperature, solar radiation, and precipitation) in China simultaneously using high spatial resolution data from 1960 to 2013. The results indicate that the daily variability of climate system dynamics (quantified by multiplying fractal dimensions of the four climatic factors) in north China was higher than that in south China. For example, the climate system dynamics were more chaotic and with higher nonlinear variation in north China, most notably in Heilongjiang Province, the major grain base of China, posing threats to food security in the context of growing national population. Spatial distribution of variability varies among different climatic factors. Our study highlights the need for a more holistic study of climate variability and trends in other countries with multiple climate types to address challenges of sustainable development.

## Introduction

Climate variability and trends have enormous influences on the environment and social development on which a growing human population relies^1–6^. Understanding climatic patterns is of great significance when many global challenges such as food insecurity, water crisis, biodiversity loss, and health issues are tied to the changing climate^7–12^.

Many studies have assessed climate trends at different spatial and temporal scales, explored the drivers that led to climate trends, or tracked the impacts of climate trends on nature and society^13–20^. For example, Hansen *et al*.^20^ analyzed the trend of global temperature change and suggested that the increasing temperature may influence sea level and the extermination of species. Sinha *et al*.^14^ studied the trend of monsoon rainfalls in South Asia over the last two millennia to explore whether climate change was caused by natural variability or anthropogenic aerosol loading^14^.

Recently, it has been recognized that the climate system has chaotic dynamics with high variability^21–25^, but trend analysis does not fully reveal these dynamics. In other words, the analyses of climate trends could only reflect the overall change of climatic factors over one period of time, but have ignored the variability of climatic dynamics to which human health, crop production and plant growth are sensitive^2, 21–26^. Variability indicates the degree of fluctuation and uncertainty of the climate change process^27^. It has great impacts on human health because the reproduction and survival rates of bacteria and viruses, which are devoid of thermostatic mechanisms, are significantly affected by temperature variability^2^. Also, climate variability has enormous influences on agricultural and economic development. Basic elements of farming – soil moisture, heat and sunlight – are affected by variability of temperature, rainfall, solar radiation, and the frequency and amplitude of extreme climate events like droughts and floods^28^. To further understand the characteristics of climatic dynamics, many studies have focused on quantifying the variability of one or two climatic factors’ long-term dynamics^22, 23, 29^. For instance, Xu *et al*.^25^ applied correlation dimension analysis in Xinjiang, China, to study the spatial-temporal variability of rainfall dynamics, and found daily variability has significant relationship with elevation. Biondi *et al*.^30^ developed tree-ring chronologies to examine decadal sea surface temperature variability in the North Pacific^30^, indicating that amplitude of variability became weak in the late l700s to mid-1800s. Morata *et al*.^31^ combined self-organizing map and multi-resolution wavelet analysis to study the variability of precipitation behaviors over the Iberian Peninsula^31^. They showed that when the variability decreases, the Iberian precipitation acts more linearly. Bodri^29^ used fractal analysis to evaluate the variability of temperature dynamics in Hungary^29^.

Among many different methods used to study the variability of climate dynamics, fractal dimensional analysis is a well-established tool for studying geophysical time series dynamics, and it has been widely adopted to analyze the variability of climate factors over time^27, 29, 32, 33^. Fractal theory allows the characteristics of variation in a given time interval to reflect the characteristics of a time interval with much finer or larger temporal resolution. Since the climate system has similar characteristics on different temporal scales (“self-similarity”)^31^, applying fractal theory to assess the variability of climate change can show a more comprehensive picture of the variability of climatic dynamics ranging from days to decades.

Despite these efforts, the comprehensive view of variability of climatic dynamics is still largely unclear due to the limited scope of climatic factors being assessed, leading to limited implications for policy and practice for agricultural and economic management^22, 23, 29^. A detailed study aimed at depicting variability of climatic dynamics that includes multiple key climatic factors simultaneously currently does not exist. Analysis that combines variability and trends of climate factors is likely to depict climatic dynamics more holistically, as it can capture characteristics of climate change with more details. Since global challenges such as food security, biodiversity loss, air pollution, water scarcity and human health are affected by the dynamics of reference evapotranspiration, temperature, solar radiation, and precipitation simultaneously^4, 34–40^, assessing their dynamics together helps reveal the nonlinearity of climatic dynamics much more holistically than studying one or two climatic factors separately. The results will reveal the implications of climate change on global challenges. Also, many important policies aimed at improving human well-being (e.g., agricultural subsidies) and environmental conservation decisions (e.g., the Natural Forest Conservation Program and Sloping Land Conversion Program in China^41–43^) are made by national governments and applied across entire nations. Thus, climate change studies at a national scale can have a more direct relevance to policy making of a country than studies at other scales (e.g., regional or global scales). Furthermore, since climatic dynamics may vary across space within one country, exploring the relationship between geographic variables (e.g., longitude, latitude, elevation) and the daily variability of climatic factor dynamics offers a more comprehensive understanding of achieving human well-being and environmental sustainability in different areas.

To address these important knowledge gaps, we selected China as a demonstration site to explore the variability and trends of the dynamics of four important climatic factors (reference evapotranspiration, temperature, solar radiation, and precipitation) simultaneously at the national scale. China is a large country with highly diverse topography, which spans climate types from southern tropical to northern boreal, western arid, eastern humid and alpine climates, resulting in a complex spatial-temporal pattern of climate conditions^44, 45^. Because many other countries have a smaller number of climate types that are experienced in China, studying patterns of climate change in China could provide useful information such as agricultural management and designing climate change adaption strategies for other countries. Based on long-term climate data from 579 meteorological stations in China, we apply fractal dimension analysis^46^ to evaluate the variability of each climatic factor and the variability of the climate system dynamics (defined as the multiplication of the four climatic factors’ fractal dimensions). Then we calculated the trend indices of the climatic factors and used the Mann-Kendall test^47^ to assess the significance of the trends of each climate factor. Finally, we studied the relationship between geographic factors (e.g. longitude, latitude, and elevation) and the variability of climatic dynamics to explore the impacts of geographic positions on the variability of climatic dynamics.

## Results

### Variability of climatic dynamics

**Figure 1:**
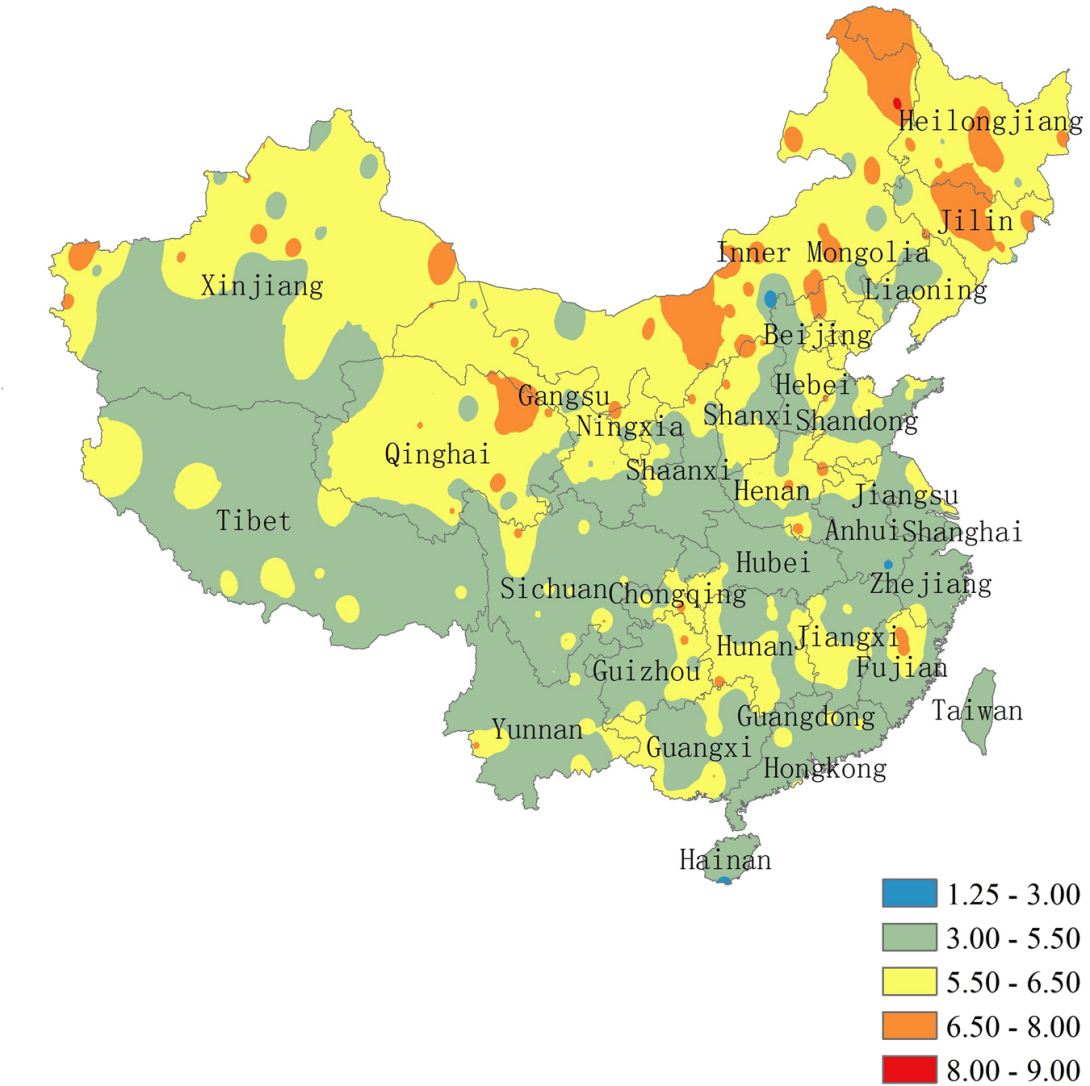
Variability of climate system dynamics in China from 1960 to 2013. The legend indicates value of fractal dimension which is used to depict variability, higher fractal dimension indicates higher variability. The map was generated by the software ArcGIS 10^81^ (http://www.esri.com/software/arcgis).

The daily variability of climate system dynamics in north China is generally larger than that in south China. The most complex climate system is found in the northeast part of China, especially in Heilongjiang and Jilin provinces (Fig. 1), two major crop production provinces in China. However, some exceptions were discovered. For example, the western part of Liaoning Province in north China has less complex climate system dynamics than the western part of Hunan Province in south China.

The monthly and seasonal variability of climate system dynamics in north China is also generally larger than in South China (Fig. 1; see Supplementary Fig. S1). And the spatial distribution of variability in China on monthly and seasonal scales is very similar to that on a daily scale.

The daily variability of climate system dynamics changes over time. From 1996 to 2013 the daily variability of climate system dynamics is lower than in previous time periods (see Supplementary Fig. S2). In all three time periods 1960–1977, 1978–1995, and 1996–2013, the variability of climate system dynamics in north China was larger than that in south China (see Supplementary Fig. S2).

**Figure 2:**
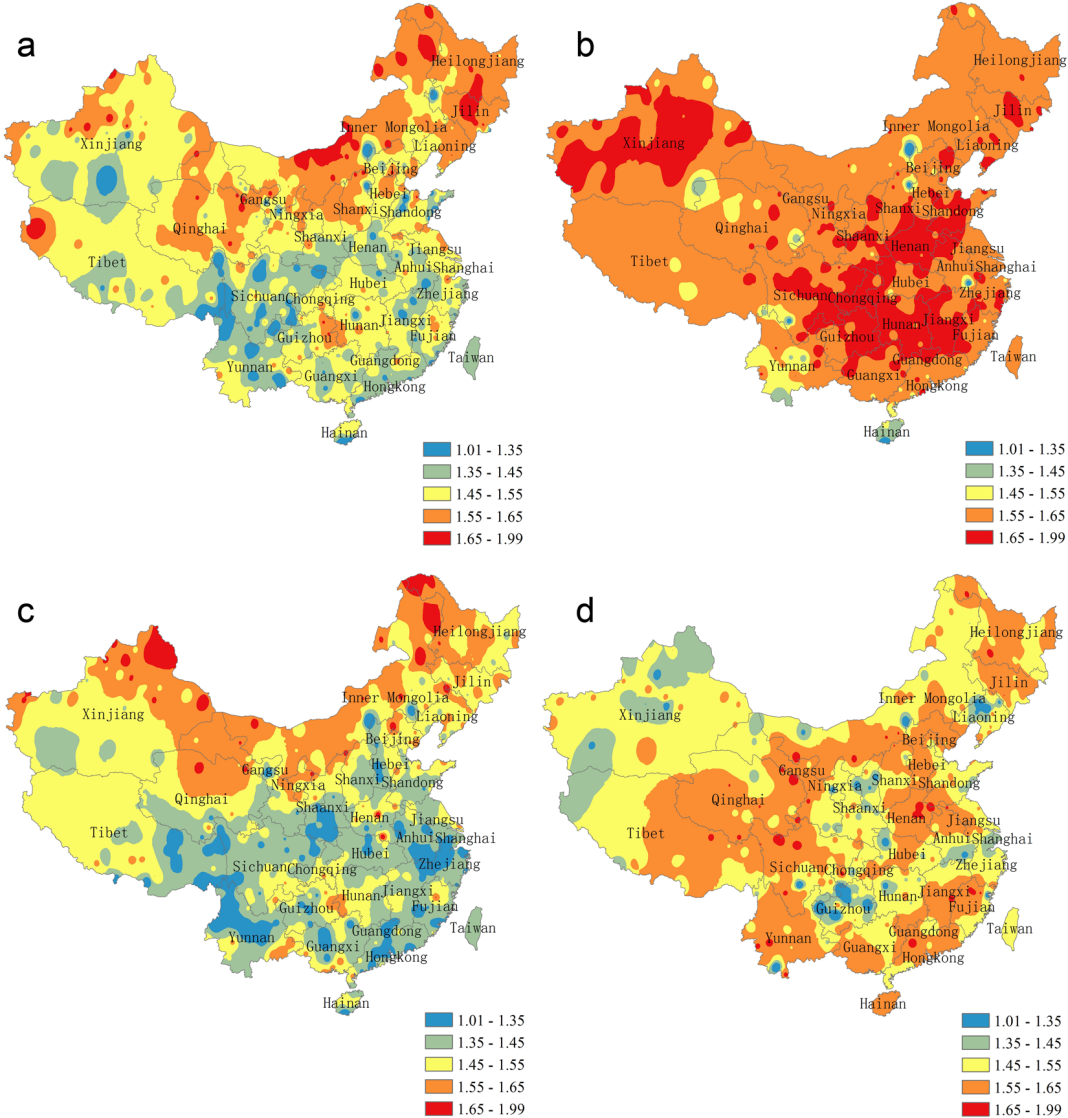
Variability of four climatic factors dynamics in China from 1960 to 2013: (**a**) ET0, (**b**) temperature, (**c**) solar radiation, (**d**) precipitation. The legend indicates value of fractal dimension which is used to depict variability, higher fractal dimension indicates higher variability. The map was generated by the software ArcGIS 10^81^.

With regard to reference evapotranspiration (ET0), its variability is higher in north China than in south China overall (Fig. 2a). The least complex ET0 dynamic is mainly distributed in southwest China, including Sichuan, Yunnan, and Chongqing, where the values of fractal dimensions are ranging from 1.01 to 1.45 (Fig. 2a). The most complex ET0 dynamic is mainly located in northeast China, as seen in Inner Mongolia and Qinghai, where the fractal dimensions are higher than 1.55 (Fig. 2a). For other areas in China, the variability of ET0 dynamic falls between these extremes (Fig. 2a).

In terms of temperature, most regions in China show high variability with fractal dimensions greater than 1.55 (Fig. 2b). South China, part of the middle and lower reaches of Yangtze River and the central area of Xinjiang, exhibit the highest variability of temperature dynamics, with fractal dimensions above 1.65 (Fig. 2b).

As for solar radiation, the variability of solar radiation dynamics in north China is higher than in south China overall (Fig. 2c). Fractal dimensions of solar radiation in most of north China are above 1.45 (Fig. 2c). Some regions in north China (e.g., Inner Mongolia, northeast Xinjiang, and northwest Heilongjiang) have fractal dimensions higher than 1.55, indicating that they have the strongest variability of solar radiation dynamics throughout China (Fig. 2c). On the other hand, fractal dimensions of solar radiation dynamics in most of south China are lower than 1.45 (Fig. 2c). Notably, southeast China has fractal dimensions lower than 1.35, which are the lowest variabilities of solar radiation dynamics in China (Fig. 2c).

Among the four factors, precipitation presents the most distinct spatial pattern compared to the other three factors. The spatial distribution of fractal dimensions of precipitation dynamics does not show substantial differences between south and north China. Yunnan, Guangdong, Jiangxi, Qinghai, Henan, Hebei and southern Inner Mongolia show higher fractal dimensions of precipitation dynamics than other areas, all that are above 1.55 (Fig. 2d).

When comparing the variability of dynamics of the four different climatic factors on a national scale, temperature dynamics had the highest variability, precipitation dynamics exhibited the second highest variability, while ET0 and solar radiation dynamics had low variability (Fig. 2a–d). The mean value of the fractal dimension of temperature, precipitation, ET0 and solar radiation among all meteorological stations are 1.62, 1.53, 1.50, and 1.47, respectively.

### Influencing factors on variability

Geographic factors have significant influences on the variability of climatic factors (Table 1). Concerning the variability of solar radiation, latitude shows a significant positive correlation while longitude demonstrates a significant negative correlation (Table 1). As for ET0’s variability, both latitude and longitude have a significant positive correlation (Table 1). For variability of temperature, elevation displays a significant negative correlation while latitude exhibits a significant positive relationship (Table 1). With regard to variability of precipitation, both elevation and longitude exhibit significant positive correlations (Table 1).

**Table 1:** Coefficients of regression model developed to evaluate the relationship between geographic factors and variability of climatic factors. (Sample size n = 579, P value is reported inside parentheses).

Regression coefficient (p value)	Latitude	Longitude	Elevation	F value in F test
Variability of solar radiation	0.0080, (0.000)***	−0.0014, (0.002)**	−6.3E-06 (0.069)	59.58, (0.000) ***
Variability of ET0	0.0069, (0.000)***	0.0011, (0.024)*	6.45E-06 (0.084)	40.50, (0.000)***
Variability of precipitation	−0.0011, (0.052)	0.0016, (0.000)***	1.5E-05, (0.000)***	8.88, (0.000)
Variability of temperature	0.00094, (0.03)*	−0.00043, (0.19)	−8.7E-06, (0.000)***	5.72, (0.000)***

Note: *** represents 0.001 significance level, ** represents 0.01 significance level, * represents 0.05 significance level.

### Climate trends

**Figure 3:**
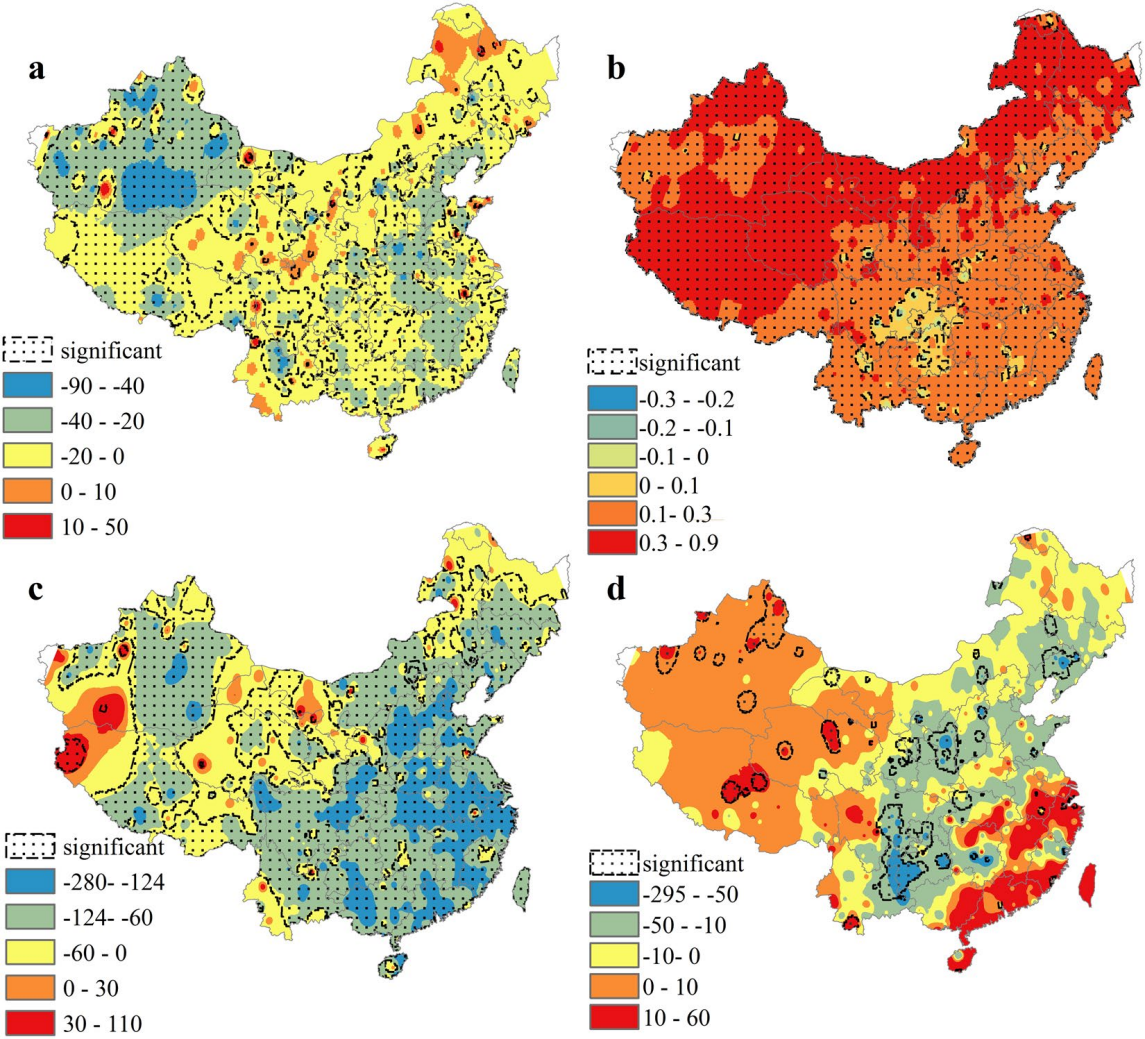
Trend of climatic factors in China from 1960 to 2013: (**a**) ET0, (**b**) temperature, (**c**) solar radiation, (**d**) precipitation. The legend indicates value of trend. The trend of climatic factors has different units: ET0 (mm/decade), temperature (°C/decade), solar radiation (MJ/(m^2^d.decade)), precipitation (mm/decade). The map was generated by the software ArcGIS 10^81^.

In order to understand dynamics of climate change at the national scale more holistically, we assess the trends of ETO, solar radiation, temperature and precipitation simultaneously in China. ET0 decreased by 0–20 mm/decade in most regions of China, while trends in east China plus Xinjiang and Tibet were significant (Fig. 3a; see Supplementary Fig. S3a). The ET0 in the middle and lower reaches of the Yangtze River and Liaoning decreased significantly at a rate of 20–40 mm/decade, with the largest rate of decrease occurring in south Xinjiang at 40–90 mm/decade (Fig. 3a; see Supplementary Fig. S3a).

Temperature rose significantly in almost all of China (Fig. 3b; see Supplementary Fig. S3b). Most regions in China had positive trends of more than 0.1 °C/decade and most areas in north China had positive trends of more than 0.3 °C/decade (Fig. 3b). Only eastern Sichuan and northern Chongqing had an insignificant increase – less than 0.1 °C/decade (Fig. 3b; see Supplementary Fig. S3b).

Solar radiation decreased over the period in most of China (Fig. 3c). In most of eastern China, Sichuan, Inner Mongolia, southern Xinjiang, and southern Tibet, the solar radiation decreased significantly at a rate of 60–124 MJ/(m^2^d.decade); decreasing trends in southeast China were more intensive, reaching a magnitude of 124–276 MJ/(m^2^d.decade) (Fig. 3c; see Supplementary Fig. S3c). Precipitation did not show significant trends in most of China, but did decrease significantly in northern Guizhou and Chongqing at a rate of more than 50 mm/decade (Fig. 3d; see Supplementary Fig. S3d). The only areas with significant increasing trends of precipitation that passed the MK test are mainly in northwest China.

## Discussion

Our analysis presents the spatial patterns of the variability and trends of four important climatic factors in China over half a century. Our findings have broad implications for socioeconomic development and the environment, such as substantial impacts on agricultural production and food security. As for the entire climate system, higher daily variability of the dynamic in north China makes it more difficult to predict climate and manage agricultural practices than in south China. Based on the integrated framework of telecoupling (socioeconomic and environmental interactions over distances)^48^, high daily variability in north China could have negative impacts on its food security, particularly in southern China where the increasing human population relies on massive food transferred from northern China^49^. This is particularly true in Heilongjiang Province, China’s major grain base^50^. There the variability of the climate system dynamics is higher than that in other provinces, yet farmers are switching from soybeans to corn, which requires a longer growing season. This choice increases the vulnerability of crop production under complex climate change^51^. Policymakers should take geographical positions into consideration when managing agricultural production under climate change on a national scale, based on their significant relationships with climatic dynamics. Increasing latitude could exacerbate the variability of climate system dynamics. The reason may be that the solar radiation angle increases as latitude increases^52^, which in China’s case leads to more intensive variability of solar radiation dynamic in the north. Also, areas in low latitude tend to have more clouds than high-latitude areas^53^, which can help decrease variability of solar radiation dynamic in south China^54^. Furthermore, since there are functional relationships existing between solar radiation and ETO^55^, higher variability of solar radiation dynamic can increase variability of ETO dynamic in north China. At the same time, more clouds in south China could also lower the variability of ETO dynamics in south China. As a result, the variability of climate system dynamics in north China is larger than in south China. Another consideration is the monitoring of climate. In China, for example, the north has generally more complex climate system dynamics than the south, yet there are more meteorological stations in the south than the north. Governments should consider deploying meteorological stations in areas with the most complex climate dynamics and weather-sensitive human interests to improve the accuracy of climate predictions and help better manage agricultural activities.

We also compared the results at the national scale derived in this study with the regional characteristics derived from previous research. The variability in regions such as northwest China, Anhui Province, Inner Mongolia, Loess Plateau and Tibetan Plateau from this study is generally higher than the corresponding variability from previous research^56–59^. The reason may be that this study has a different temporal scale compared to previous studies (daily vs. annual time steps), and thus revealing characteristics of daily scale analysis based on fractal theory could detect more variation and uncertainty than previous research. Temperature daily variability in our results is approximately 25%~33% higher than the corresponding temperature annual variability in previous studies at the regional level, while precipitation in our results is 5%~8% higher than the precipitation annual variability in previous studies, but the difference in temporal scale can explain almost all the differences. Taking the previous study about Anhui Province as one example (because that study has the same dataset and similar time duration which is from 1955 to 2011), the daily variability of temperature in Anhui Province from our results is approximately 32.9% higher than the annual variability of temperature in that study, while the daily variability of precipitation is 6.2% higher. However, after we recalculated the annual variability based on our results and data, we found the annual variability of temperature from our results is only 2% lower than that in the previous study, while precipitation is only 1.1% higher than the previous result. So the difference in temporal scale can account for most of the differences in variabilities. We also found the regions with relatively higher variability in previous studies generally showed a relative higher variability in our results. For example, the temperature annual variability of the Tibet Plateau is higher than that in Anhui Province in previous studies, and in our study the daily temperature variability in the Tibet Plateau is also higher than it in Anhui Province. On the other hand, the variability at a daily scale is similar with variability at monthly and seasonal scales (Fig. 1; see Supplementary Fig. S1). This is likely because fractal theory can reflect self-similarity of climate dynamics across multiple temporal scales such as daily scale, monthly scale and seasonal scale given enough time series data, while data in previous research at annual scale is too limited for fractal theory to reflect variability of climatic dynamics. Therefore, we suggest that future studies about variability should focus more on daily, monthly and seasonal scales instead of annual scale.

Our findings about the trend of temperature change differ slightly from previous results. Temperature has increased more than 0.3 °C/decade in most areas in north China and 0.1–0.3 °C/decade in most of south China, which is slightly higher than results reported by Qian and Qin (2006)^60^. The reason may be that our study covers 1960–2013, 13 years longer than 1960–2000 covered by Qian and Qin (2006)^60^, and global warming has continued to accelerate after the year 2000. Only the eastern part of Sichuan basin and western part of Chongqing show insignificant temperature trends, which are lower than 0.1 °C/decade. It is likely due to the decreasing sunlight, increasing cloud cover and large amounts of aerosols in the Sichuan Basin, which could mitigate the increase of temperature and even decrease surface temperature^61, 62^. The second reason is that the Sichuan Basin is the only oceanic climate area in inland China, surrounded by the mountains of Qinghai-Tibet Plateau and Himalayas. They provide a natural barrier to airflows like monsoons, thus helping regulate local temperature change. The relationships between geographic factors and the variability of temperature dynamics are less significant. Higher variability exists in the temperature dynamics than in the dynamics of other climatic factors, but the variance in spatial distribution of variability of temperature dynamics is smaller than in other climatic factors. The reason may be that temperature dynamics are potentially affected by some factors outside of China on a larger scale, such as El Niño^63^. Thus, government agencies should not only consider mechanisms within the country, but also consider impacts from global scale in this telecoupled world^64^. Factors like the East Asian monsoon, El Niño phenomenon, and sunspot activity may also affect the variability of the climate system dynamics in China. The variability of the East Asian monsoon brings changes in the moist air and heat brought to China, which could affect the variability of temperature and precipitation dynamic in China^65, 66^. El Niño-Southern Oscillation shapes the variation of sea surface temperatures and wind in the tropical eastern Pacific Ocean^67^, which may influence the variability of precipitation and temperature dynamic in China through teleconnection between climate systems^68^. Periodic sunspot activity could have an impact on the variability of solar radiation, temperature and ETO dynamic in China.

Our study confirms the self-affine characteristics of climate system dynamics, which means the measurements taken at different time scales have similar statistical characteristics. Almost all fractal dimensions for each climate factor time series are between 1.35 and 1.65 (Fig. 2), indicating fractal phenomenon and self-affine time series. We also found the distribution of variability of climate system dynamics at a daily scale in China is similar to that at the monthly and seasonal scales (Fig. 1; see supplementary Fig. S1).

This study presents the spatial patterns of the variability and trends of climate change in a nation over half a century and reveals its implications for sustainability. However, how variability of climatic dynamics affects human adaptation to climate change and how external factors from global scales influences variability of climatic dynamics within the country are still unknown. Based on the integrated telecoupling framework^48^, future research should incorporate socioeconomic factors (e.g., GDP, human population dynamics) to explore how variability of climatic dynamics interacts with human societies across multiple scales, which may help make more comprehensive and well-informed national and international decisions and policies in the pursuit of the sustainability of coupled human and natural systems under complex climate change.

## Methods

**Figure 4:**
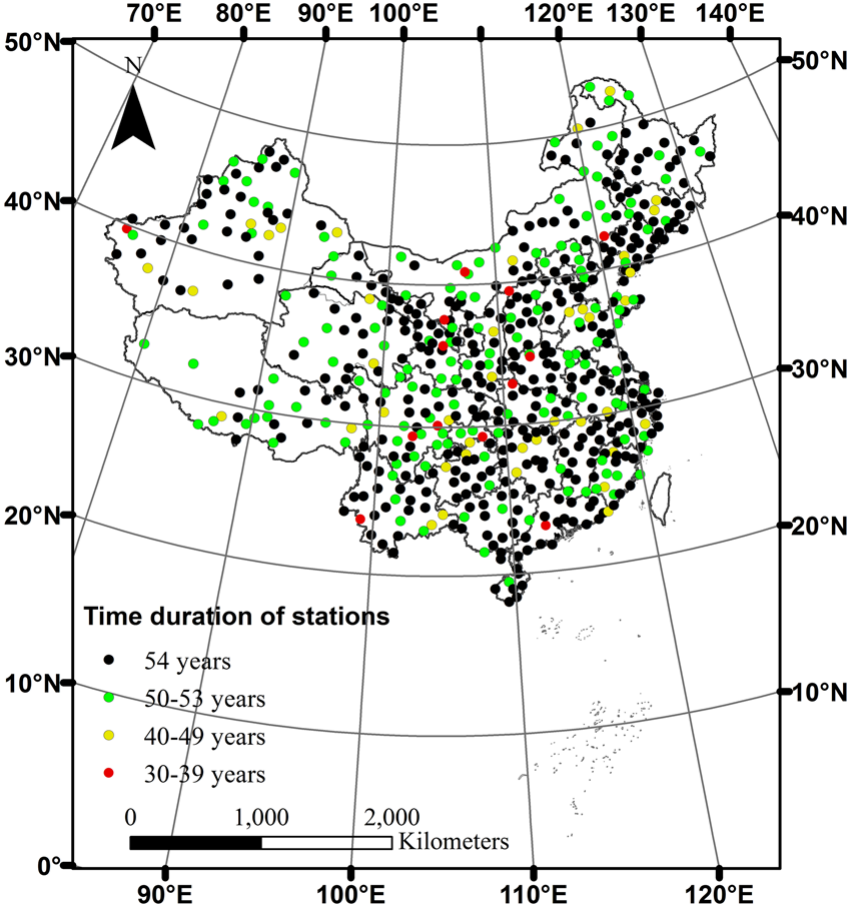
Spatial distribution of national meteorological stations in China and their data durations. Data source: China Meteorological Data Sharing System http://cdc.cma.gov.cn. The map was generated by the software ArcGIS 10^81^.

We obtained climate time series data from 756 meteorological stations in China. After an initial quality check, the long-term climate data from 579 meteorological stations in China were derived from the daily data set of the China Meteorological Data Sharing System (http://cdc.cma.gov.cn). For all observational time series, we excluded the years with missing data more than 20 days in total or years with more than 10 consecutive days of missing data. To fill in small data gaps (≤10 days) found in some years, a simple linear interpolation algorithm was performed. Stations with less than 30 consecutive years of data were excluded. Among the 579 stations (Fig. 4), 378 stations have complete daily data for 54 years (from 1960 to 2013), while the other 201 stations have data with durations ranging from 30 to 53 years.

We calculated the fractal dimensions and trend indices of each climatic factor from 1960–2013 in the 579 meteorological stations and performed Mann-Kendall tests for trends. After deriving the indices of climatic factors, we used the Inverse Distance Weighted Interpolation (IDW) tool in ArcGIS to derive national maps^69^. Inverse Distance Weighted Interpolation (IDW) is one of the most commonly used interpolation methods because of its simplicity and relatively high accuracy^70, 71^. IDW computes the value for a given location based on existing values surrounding it. The measured values located closer to the prediction location have stronger influences on the predicted value. It has been widely used to interpolate distribution of the dynamics of various climate factors^71–73^. In order to check its effectiveness, we compared its accuracy with other methods including Ordinary Kriging and Spline by comparing Root Mean Square Error (RMSE) between real fractal dimensions in the stations and the corresponding interpolated fractal dimensions. Using the interpolation of the distributions of seasonal fractal dimensions during 1960–2013 in China as an example, we calculated RMSE from IDW, Ordinary Kriging, and Spline and found values of 0.03, 0.67 and 0.09, respectively. The IDW exhibited the lowest interpolation error among three methods, and thus chosen as our interpolation method.

Fractal theory has been applied in many fields to study the complexity of systems, such as financial markets and soil structure^74, 75^. Recently, it has been used to study the variability of climatic dynamics. Fractal dimension D of climate time series can be derived from Hurst index of meteorological parameters^76^. We derive Hurst index of climate time series from R/S (rescaled range analysis), which is a prediction method in nonlinear science presented by Hurst and developed into fractal theory for studying time series by Mandelbrot^77, 78^. The basic principle of the R/S method is described below. For particular climate factor’s time series *t* = *1*, *2*, …, *n*, *x*(*t*) indicates the value of the climate factor at time t, and the mean sequence *x*_*τ*_ for any integer *τ* is defined as:

1 $${x}_{\tau }=\frac{1}{\tau }\sum _{t=1}^{\tau }x(t),\tau =1,2,\mathrm{...},n$$

Cumulative deviation *X*(*t,τ*) is calculated:

2 $$X(t,\tau )=\sum _{k=1}^{t}[x(k)-{x}_{\tau }],1\le t\le \tau $$

Range sequence *R*(*τ*) is calculated:

3 $$R(\tau )=\,{\rm{\max }}\,X(t,\tau )-\,{\rm{\min }}\,X(t,\tau ),t=1,2,\mathrm{...},n$$

Then a usual standard deviation estimator *S*(*τ*) is applied:

4 $$S(\tau )=\sqrt{\frac{1}{\tau }\sum _{t=1}^{\tau }{[x(t)-{x}_{\tau }]}^{2}}$$

For the ratio *R/S* = *R*(*τ*)/*S*(*τ*), if the following relationship exists:

5 $$R/S\propto {\tau }^{H}$$

then the Hurst phenomenon exists in the climate factor’s time series *{x(t)}, t* = *1, 2,…, n*. Next, we applied a log transformation to the equation (5), so that *log(R/S)* has linear relationship with *log(τ)*. *H* is then the only linear coefficient, and can be calculated using the least square method. The *H* is the Hurst index. Feder *et al*.^76^ verified the relationship between the fractal dimension *D* of a time series and its Hurst index *H*, which is represented by equation (6)^76^:

6 $$D=2-H$$

D ranges from 1 to 2. Higher D of time series of climate factors means higher variability of the climate factor dynamic, which indicates the climate factor changes with higher frequency and more uncertainty.

In order to assess the variability of climate system dynamic which consists of four climatic factors dynamics together, we define the variability of climate system dynamic *D*_*climate system*_ as the multiplication of fractal dimensions of four climatic factors dynamics:

7 $${D}_{climatesystem}={D}_{ET0}\ast {D}_{temperature}\ast {D}_{solarradiation}\ast {D}_{precipitation}$$

Trend ${\rm{\beta }}$ was calculated by using equation (8)^79^:

8 $$\beta =median[\frac{{X}_{j}-{X}_{i}}{j-i}],\forall i < j$$

The *X*_*i*_ and *X*_*j*_ are the climate time series for one climatic factor; i and j represent particular days i and j in time period, respectively. We used the Mann-Kendall trend test for the significance of the trends of climatic factors^47, 80^.

## Electronic supplementary material

Supplementary Information
