# Supplementary material for: Climate variability and trends at a national scale
Source: Sci Rep. 2017 Jun 12;7:3258. doi: 10.1038/s41598-017-03297-5 (PMC5468283; doi:10.1038/s41598-017-03297-5)
Supplement: Supplementary file 1 — Supplementary Information [file 41598_2017_3297_MOESM1_ESM.doc]

**Climate variability and trends at a national scale**

Zhenci Xu, Ying Tang, Thomas Connor, Dapeng Li, Yunkai Li, Jianguo Liu


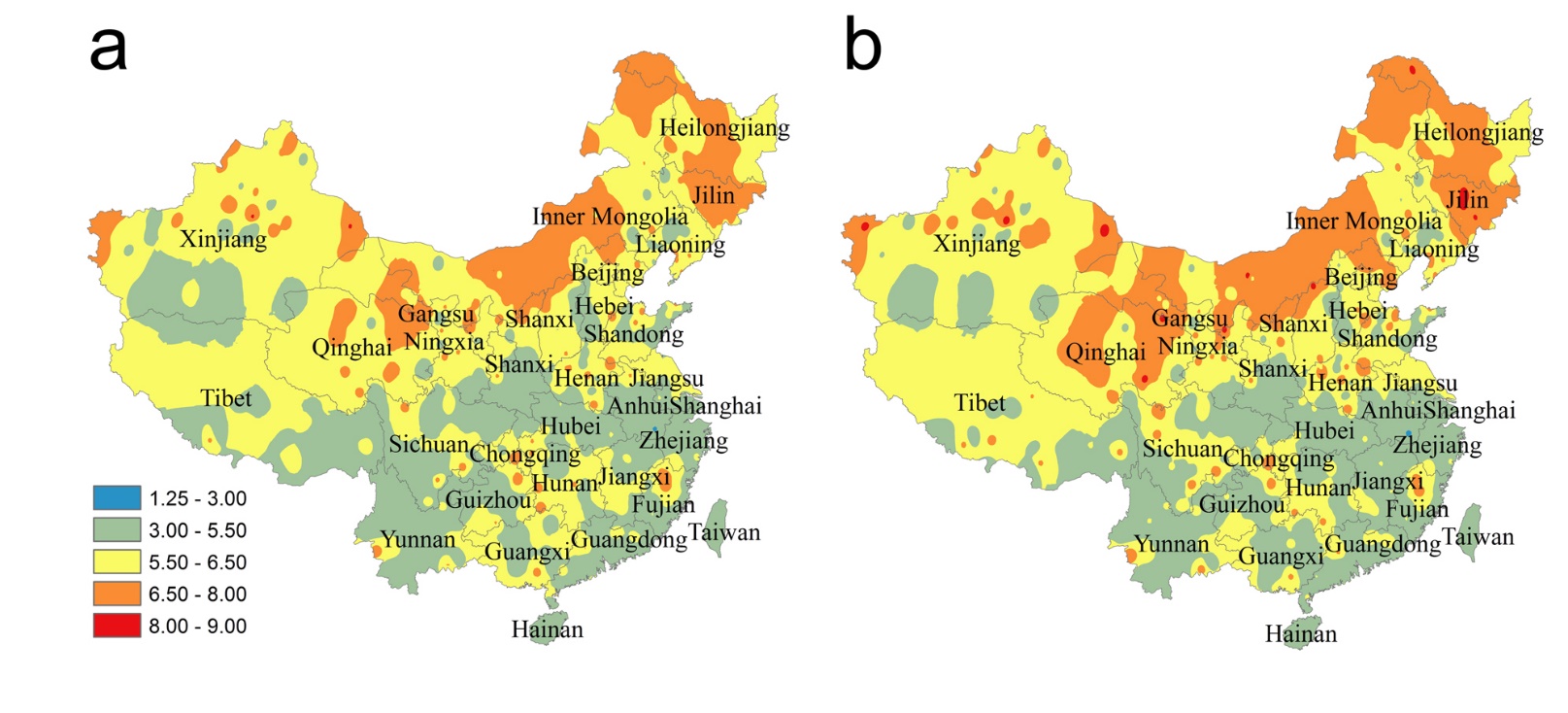
Supplementary Fig. S1 Variability of climate system dynamics in China from 1960-2013 at different temporal scales: a. monthly scale, b. seasonal scale. The legend indicates value of fractal dimension which is used to depict variability, higher fractal dimension indicates higher variability. The map was generated by the software ArcGIS 10^1^ (<http://www.esri.com/software/arcgis>).


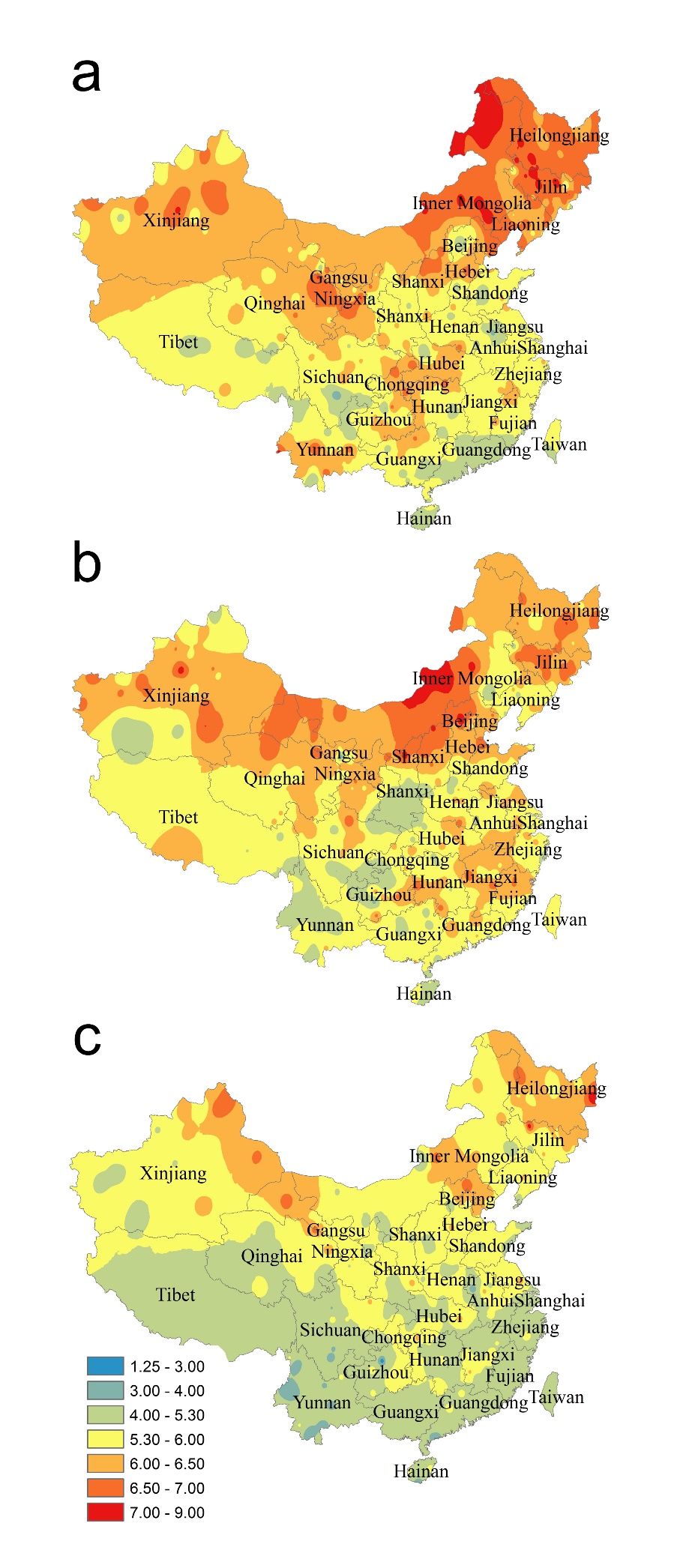


Supplementary Fig. S2 Variability of climate system dynamics in China from three periods: a. 1960-1977, b. 1978-1995, c. 1996-2013. The legend indicates the value of fractal dimension used to depict variability, higher fractal dimension indicates higher variability. The map was generated by the software ArcGIS 10^1^ (<http://www.esri.com/software/arcgis>).


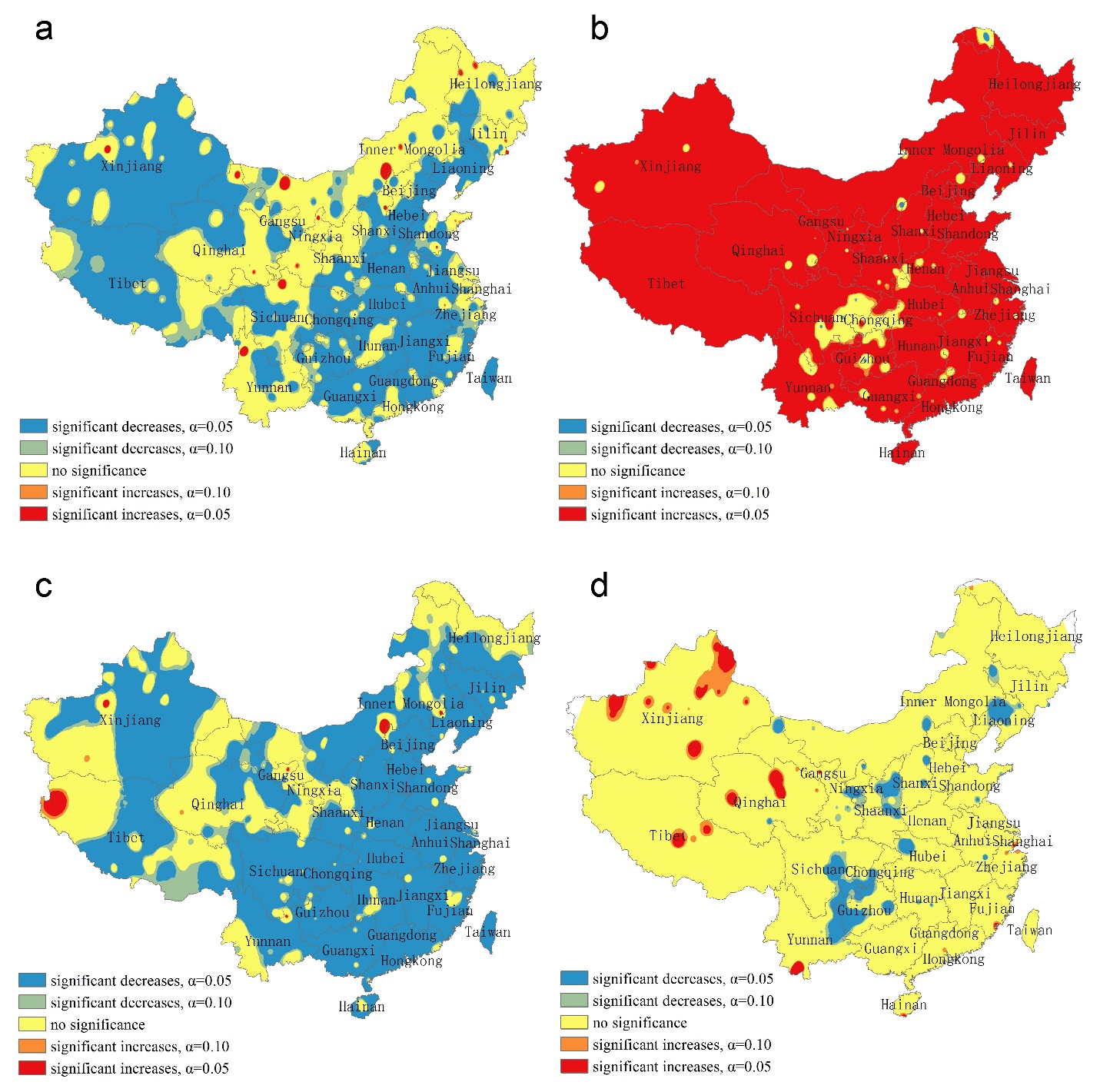


Supplementary Fig. S3 Map of significant test of climate factors trend in China: a. ET0, b. temperature, c. solar radiation, d. precipitation. The legend indicates the result of significant test. The map was generated by the software ArcGIS 10^1^ (<http://www.esri.com/software/arcgis>).

**Supplementary Reference**:

1 ESRI, R. ArcGIS desktop: release 10*. Environmental Systems Research Institute,* CA (2011).
